# P21 Evidence of impact of national stewardship tools for secondary care in England

**DOI:** 10.1093/jacamr/dlae217.025

**Published:** 2025-01-23

**Authors:** R Berry, D Ashiru-Oredope

**Affiliations:** UK Health Security Agency, London, UK; UK Health Security Agency, London, UK

## Abstract

**Background:**

Antimicrobial resistance (AMR) is recognized nationally and internationally as a major risk to public health. Inappropriate use of antibiotics is a driver, and good antimicrobial stewardship (AMS) is a key factor in mitigating this risk. Since 2011, the UK Health Security Agency (UKHSA) and its predecessor organizations have developed national tools to support AMS in secondary care. These include tools that are secondary care-specific e.g. Start Smart-Then Focus (SSTF) toolkit, as well as some to support AMS across multiple sectors e.g. Antibiotic Guardian (AG) campaign. It is important to ascertain whether these tools have had impact to inform ongoing work.

**Objectives:**

To identify and assess the evidence of impact of national AMS tools developed by UKHSA that are used in secondary care in England.

**Methods:**

A scoping review was conducted. Literature database (PubMed), grey literature search (Policy Commons), English Surveillance Programme for Antimicrobial Utilisation and Resistance (ESPAUR) reports (2014–2024), UK AMR Strategy (2013–2018), National Action Plan (2019–2024 and 2024–2029), UK 20 year AMR vision, NICE guidelines and NHS England policy documents were reviewed. Data were collected on number of citations of paper on tool development, number of views of the host webpage (2020–2024), and evidence of use in the UK and reference to the tool internationally.

**Results:**

Twelve tools were identified. Peer-reviewed publications for development were available for nine, with an average citation rate of 44 (range 2–206). Website analytics were available for the host website of seven tools, with an average of 71 588 visits in a 4 year period (range 1526–407 761). Three tools showed evidence of impact through published articles on PubMed with 32 articles identified (excluding development papers). Six tools showed evidence of impact when reviewing grey literature, with 527 articles identified, 74 of which were from international organizations. Nine tools were mentioned in UK strategy papers on AMR, NICE guidance or within NHSE policy documents. Four tools had evidence of international impact, with evidence from countries across all continents. The tools with most evidence of impact were the SSTF Toolkit and the AG Campaign. The tools with least evidence of impact were the National Audit Tool for Secondary Care and the Secondary Care Peer-to-peer Review Tool.

**Conclusions:**

There is evidence that the UKHSA AMS tools developed to support secondary care or to support multiple sectors have had wide-ranging impact. This includes impact on hospitals and government departments in England and the devolved nations, and on UK organizations such as Royal Colleges and charities. They have impacted guidance and policy through being referenced in UK AMR action plans and NICE guidance. There is also evidence of global international impact. Some tools have more evidence of impact than others, in particular those that are available on websites, although a limitation of the study is that a full process evaluation was not carried out. Future work will include surveying the AMS workforce to determine which tools are used in secondary care, and to consider further knowledge mobilization activities to support ongoing impact.

